# International Clinics

**Published:** 1902-09

**Authors:** 


INTERNATIONAL CLINICS. A quarterly of clinical lectures and especially prepared articles of medicine, neurology, surgery, therapeutics, obstetrics, pediatrics, pathology, dermatology, diseases of the eye, ear, nose and throat and other topics of interest to students and practitioners. By leading members of the medical profession throughout the world. Edited by Henry W. Cattell, A. M., M. D., Philadelphia; with the collaborations of John B. Murphy, M. D.; Alex. D. Blockader, M. D., Chicago; H. C. Wood, M. D., Philadelphia; T. M. Rotch, M. D.; E. Landolt, M. D.; T. G. Morton, M. D., Philadelphia; Chas. H. Reed, M. D., Philadelphia; J. W. Ballantyne, M. D., of Edinburgh, and John Harold, of London. With regular correspondents in Montreal, London, Paris, Leipsic, and Vienna. Volume I. Eleventh Series. 1901. Pages, 312. Price, $2.00. Philadelphia: J. B. Lippincott Company. 1901.

Under the heading "Therapeutics" the twenty-six pages of this volume are devoted to notes on new remedies by A. A. Stevens, M. D.; the treatment of chronic gonorrhea, or gleet, by Alexander Renault, M. D.; and the treatment of eczema, by Professor H. Renault.

Then follow under the head of "Medicine," sacculated pleurisy, by Delafield; a report of one hundred cases of aortic anuerism, by Batty Shaw; the normal temperature range, by Marx; scarlet fever, by Herbert Biss; gonorrheal rheumatism, by Campbell Williams, and the pneumonia of influenza and its treatment, by Solis-Cohen.

Prichard, under the head of "Neurology," contributes an excellent article on the nervous diseases and psychoses following la grippe.

Under the head of "Surgery" a large and varied number of subjects are discussed by such men as Rodman, Roncali, Elsberg and Holmes.

The same thorough treatment of other subjects follow.

